# Factors Associated With Veterans Use of Community vs VA Emergency Departments

**DOI:** 10.1001/jamanetworkopen.2025.43062

**Published:** 2025-12-08

**Authors:** Anita A. Vashi, Steven M. Asch, Tracy Urech, Siqi Wu, Linda D. Tran

**Affiliations:** 1 Center for Innovation to Implementation, VA Palo Alto Health Care System, Menlo Park, California; 2 Department of Emergency Medicine, University of California, San Francisco; 3 Department of Emergency Medicine (Affiliated), Stanford University, Stanford, California; 4 Stanford Primary Care and Population Health, Stanford University, Stanford, California; 5 Health Economics Resource Center, VA Palo Alto Health Care System, Palo Alto, California; 6 Surgery Policy Improvement and Education Center, Stanford Medicine, Stanford University, Stanford, California

## Abstract

**Importance:**

The US Department of Veterans Affairs (VA) aims to ensure all veterans have access to emergency care, whether at VA or community facilities. However, the factors influencing where veterans seek care remain poorly understood.

**Objective:**

To assess factors associated with veterans use of community vs VA EDs.

**Design, Setting, and Participants:**

This retrospective cross-sectional study included all veterans aged 18 years or older with at least 1 VA or community ED visit across the contiguous US and Puerto Rico between October 1, 2021, and September 30, 2022. Data were analyzed from October 3, 2024, to March 10, 2025.

**Exposures:**

Patient demographics, clinical characteristics, prior VA and community health care use, differential distance to the nearest VA and community EDs, and facility complexity.

**Main Outcomes and Measures:**

Community or VA ED visit. Logistic regression models were used to assess factors associated with community ED use compared with VA ED use.

**Results:**

The sample included 2 777 564 ED visits by 1 359 850 veterans (mean [SD] age, 61.9 [16.1] years; 1 202 964 [88.5%] male). Of these visits, 1 000 437 (36.0%) occurred at community EDs. Community ED use was associated with greater differential ED distance (>64.0 km: adjusted odds ratio [AOR], 16.20; 95% CI, 15.96-16.44), prior community ED use (≥5 prior visits: AOR, 17.87; 95% CI, 17.14-18.63), and high-acuity diagnoses (eg, cardiac arrest and ventricular fibrillation: AOR, 84.57, 95% CI, 65.47-109.25). Prior VA ED visits (1 prior visit: AOR, 0.28; 95% CI, 0.28-0.28) and primary care use (AOR, 0.78; 95% CI, 0.77-0.79) were associated with lower odds of community ED use. Racial and ethnic minority veterans were consistently less likely to use community EDs compared with White veterans (Asian veterans: AOR, 0.64 [95% CI, 0.61-0.67]; Black veterans: AOR, 0.76 [95% CI, 0.76-0.77]; and Hispanic veterans: AOR, 0.73 [95% CI, 0.72-0.74]).

**Conclusions and Relevance:**

In this cross-sectional study, community ED use among veterans was associated with geographic access, prior care patterns, and diagnosis acuity. Targeted strategies appear to be needed to strengthen care coordination and ensure equitable emergency care access.

## Key Points

**Question:**

What factors are associated with veterans use of community emergency departments (EDs) vs US Department of Veterans Affairs (VA) EDs?

**Findings:**

In this cross-sectional study of 2 777 564 ED visits by veterans, community ED use was associated with greater differential distance, prior community ED use, and high-acuity diagnoses. Female, unhoused, and White veterans were more likely to use community EDs, while prior VA engagement and proximity to high-complexity VA facilities were associated with lower odds of community ED use.

**Meaning:**

These findings suggest that geographic access, patterns of prior care, and demographic factors are associated with veterans’ ED use, highlighting opportunities to strengthen VA and community care coordination and improve equitable access to emergency services.

## Teaser

This cross-sectional study assesses patient, encounter, and facility factors associated with use of US Department of Veterans Affairs (VA) vs community emergency departments among veterans.

## Introduction

The US Department of Veterans Affairs (VA) provides health care to more than 9 million veterans through its own facilities and by purchasing services from non-VA organizations, a system known as community care. Reliance on community care has expanded markedly in recent years, sparking debate over how best to balance investment in the VA’s infrastructure with reliance on external organizations.^1^ Supporters highlight expanded access and convenience, particularly for veterans living far from VA facilities, while critics point to higher costs, fragmented care, and concerns about weakening the VA’s capacity.^2^

Emergency care has become a focal point in this debate. Since the 1950s, US Congress has authorized the VA to purchase emergency services in community settings,^3^ and today these services play a critical role in ensuring timely access for veterans living far from VA facilities. As reliance on community care has grown, policy attention has shifted from expanding access to optimizing how community emergency care is integrated into the VA system. While community care offers important advantages in acute care contexts, it also presents challenges related to continuity, oversight, and coordination.^2^ Prior studies comparing VA and non-VA acute care have even suggested potential advantages of VA facilities in outcomes and quality.^4^ Emergency care is now the largest category of VA community care spending, reflecting both its central role in acute access and the rapid growth of community ED use. ED visits are costly, often represent patients’ most immediate health needs, and frequently serve as an entry point to further treatment.

Despite the large and growing role of community EDs in caring for veterans, little is known about which factors are associated with where veterans receive emergency care. Prior qualitative work has highlighted the importance of distance, perceived acuity, and financial considerations in shaping veterans’ decisions,^5,6^ but quantitative evidence is limited. To address this gap, we conducted a national analysis of factors associated with veterans use of VA and community EDs, with the goal of providing descriptive evidence that may inform policy discussions, resource planning, and strategies to support coordinated, veteran-centered emergency care.

## Methods

### Data Sources

We conducted a retrospective cross-sectional study using data from the VA Corporate Data Warehouse, which aggregates clinical and administrative data across VA systems. VA ED visits were identified from the Corporate Data Warehouse, while VA-paid community ED visits were obtained from the Office of Integrated Veteran Care. Primary diagnoses were categorized using the Agency for Health Care Research and Quality Clinical Classifications Software Refined for *International Statistical Classification of Diseases, Tenth Revision, Clinical Modification* codes.^7^

The study sample included all VA and community ED visits between October 1, 2021, and September 30, 2022 (fiscal year 2022), among veterans aged 18 years or older residing in the contiguous US or Puerto Rico. Each ED visit was treated as an independent observation. When multiple ED visits occurred on the same day, one was selected at random. The Stanford University institutional review board determined that the evaluation did not meet the federal definition of human participants research and did not require approval or informed consent. The study followed the Strengthening the Reporting of Observational Studies in Epidemiology (STROBE) reporting guideline.

### Measures

The primary outcome was ED site of care (VA vs community). The independent variables represented factors that we hypothesized would be associated with that outcome: condition acuity, health status, social risk factors, recent VA care engagement, VA facility complexity, and geographic proximity.

Acuity was proxied by Agency for Health Care Research and Quality Clinical Classifications Software Refined diagnosis categories (≥1000 visits per category). Health status included age, sex, and Nosos score (a VA risk adjustment measure, where >1.00 reflects greater-than-average health care costs).^8^ Social risk factors included housing instability, race (American Indian or Alaska Native, Asian, Black or African American, Native Hawaiian or Other Pacific Islander, or White), ethnicity (Hispanic or Latino or non-Hispanic or non-Latino), and VA priority group (which reflects service-connected disability, income, and co-pay eligibility).^9^ Race and ethnicity were obtained from VA electronic health record data (Corporate Data Warehouse) and standardized via the Observational Medical Outcomes Partnership common data model; values are primarily self-reported, with supplemental entry by proxies or staff. We included race given racial disparities in health care.

Recent VA care engagement was defined by primary care or mental health care use in the prior 12 months and nurse advice line contact as recently as 48 hours prior to the visit. Geographic proximity from each veteran’s residence to the nearest VA and community EDs was calculated using the Haversine formula. Differential ED distance was calculated as the absolute difference between the distance to the nearest VA ED and the distance to the nearest community ED and then categorized into 5 groups: 0 to 8, 9.6 to 16.0, 17.6 to 32.0, 33.6 to 64.0, and greater than 64.0 km. In most cases, these groups reflected the additional distance a veteran would need to travel to reach a VA ED compared with a community ED. VA facility complexity was defined using the VA’s national classification system (levels 1a to 3, with 1a being the most complex), based on service availability, intensive care unit capacity, and patient mix.^10^

### Statistical Analysis

We summarized patient, encounter, and facility characteristics by ED location using standardized mean differences. Multivariable logistic regression models estimated the associations between patient, encounter, and facility factors and the likelihood of receiving care at a community ED compared with a VA ED. Average marginal effects (AMEs) were calculated to aid interpretation. All models were adjusted for temporal variables (month and day of the week) and used robust SEs to account for clustering by veteran. Analyses were conducted using Stata, version 17.0 (StataCorp) from October 3, 2024, to March 10, 2025. Two-sided *P* < .05 indicated statistical significance.

To assess the robustness and context of associations across different care-seeking scenarios, we estimated 4 models tailored to specific veteran subgroups. These models were selected to isolate distinct factors associated with community ED use. Model 1 included all ED visits (baseline associations [n = 2 777 564]); model 2, ED visits made by veterans for whom their differential ED distances were 8.0 km or less (veterans with comparable geographic access to address distance-based differences [n = 838 850]); model 3, chest pain ED visits among veterans with differential ED distances of 8.0 km or less (a common ED presentation [n = 32 279]); and model 4, ED visits among veterans with no ED use in the 12 months before their visit (to isolate factors associated with initial ED site choice among those without recent emergency care exposure [n = 849 477]).

## Results

We excluded 30 818 visits in which a veteran accessed both a VA ED and a community ED on the same day to focus on site-level decision-making. The final sample included 2 777 564 ED visits by 1 359 850 unique veterans in fiscal year 2022 (mean [SD] age, 61.9 [16.1] years; 156 886 female [11.5%]; 1 202 964 male [88.5%]). Of these visits, 1 000 437 (36.0%) occurred at community EDs and 1 777 127 (64.0%) at VA EDs. A total of 24 667 visits (0.9%) were by American Indian or Alaska Native patients; 19 930 (0.7%), Asian patients; 688 892 (24.8%), Black patients; 21 975 (0.8%), Native Hawaiian or Other Pacific Islander patients; 1 857 296 (66.9%), White patients; and 164 804 (5.9%), patients with unknown race. In addition, 214 456 visits (7.7%) were by Hispanic patients; 2 501 201 (90.1%), non-Hispanic or non-Latino patients; and 61 907 (2.2%), patients with unknown ethnicity. Table 1 presents patient and facility characteristics overall and stratified by ED location. In the study sample, 2 624 523 veterans (94.5%) lived closer to a community ED than a VA ED.

**Table 1.** Characteristics of VA ED Visits and Community ED Visits Purchased by the VA in Fiscal Year 2022

Characteristic	Visits, No. (%)			SMD
	Overall (N = 2 777 564)	VA ED (n = 1 777 127)	Community ED (n = 1 000 437)	
Age group, y				
18-44	478 058 (17.2)	315 792 (17.8)	162 266 (16.2)	0.114
45-64	853 840 (30.7)	572 431 (32.2)	281 409 (28.1)	
≥65	1 445 666 (52.0)	888 904 (50.0)	556 762 (55.7)	
Sex^a^^,^^b^				
Female	312 851 (11.3)	204 977 (11.5)	107 874 (10.8)	0.024
Male	2 464 713 (88.7)	1 572 150 (88.5)	892 563 (89.2)	
Race^a^^,^^c^				
American Indian or Alaska Native	24 667 (0.9)	14 867 (0.8)	9800 (1.0)	0.348
Asian	19 930 (0.7)	15 807 (0.9)	4123 (0.4)	
Black or African American	688 892 (24.8)	529 483 (29.8)	159 409 (15.9)	
Native Hawaiian or Other Pacific Islander	21 975 (0.8)	14 905 (0.8)	7070 (0.7)	
White	1 857 296 (66.9)	1 096 754 (61.7)	760 542 (76.0)	
Unknown	164 804 (5.9)	105 311 (5.9)	59 493 (5.9)	
Ethnicity^a^^,^^c^				
Hispanic or Latino	214 456 (7.7)	158 947 (8.9)	55 509 (5.5)	0.132
Not Hispanic or Latino	2 501 201 (90.1)	1 580 442 (88.9)	920 759 (92.0)	
Unknown	61 907 (2.2)	37 738 (2.1)	24 169 (2.4)	
Unhoused	296 687 (10.7)	211 645 (11.9)	85 042 (8.5)	0.113
Rurality				
Urban	194 008 (70.0)	1 392 283 (78.3)	551 725 (55.1)	0.508
Rural	833 556 (30.0)	384 844 (21.7)	448 712 (44.9)	
VA priority group^d^				
High disability	1 539 062 (55.4)	977 948 (55.0)	561 114 (56.1)	0.039
Low to moderate disability	483 534 (17.4)	314 821 (17.7)	168 713 (16.9)	
Low income	484 299 (17.4)	305 999 (17.2)	178 300 (17.8)	
No disability	270 669 (9.7)	178 359 (10.0)	92 310 (9.2)	
Nosos risk score^e^				
<1.00	1 006 826 (36.2)	597 628 (33.6)	409 198 (40.9)	0.175
1.00-1.99	983 005 (35.4)	633 036 (35.6)	349 969 (35.0)	
≥2.00	787 733 (28.4)	546 463 (30.7)	241 270 (24.1)	
VA primary care use in past 12 mo				
Yes	2 643 909 (95.2)	1 703 670 (95.9)	940 239 (94.0)	0.086
No	133 655 (4.8)	73 457 (4.1)	60 198 (6.0)	
VA mental health use in past 12 mo				
Yes	1 354 923 (48.8)	900 275 (50.7)	454 648 (45.4)	0.105
No	1 422 641 (51.2)	876 852 (49.3)	545 789 (54.6)	
VA advice line use in past 2 d				
Yes	345 466 (12.4)	221 054 (12.4)	124 412 (12.4)	<0.001
No	2 432 098 (87.6)	1 556 073 (87.6)	876 025 (87.6)	
Community ED visits in past 12 mo				
0	1 942 582 (69.9)	1 486 680 (83.7)	455 902 (45.6)	0.892
1	398 778 (14.4)	173 942 (9.8)	224 836 (22.5)	
2	172 187 (6.2)	55 249 (3.1)	116 938 (11.7)	
3	90 389 (3.3)	24 056 (1.4)	66 333 (6.6)	
4	52 566 (1.9)	12 470 (0.7)	40 096 (4.0)	
>5	121 062 (4.4)	24 730 (1.4)	96 332 (9.6)	
VA ED visits in past 12 mo				
0	1 308 277 (47.1)	547 501 (30.8)	760 776 (76.0)	1.038
1	501 116 (18.0)	391 627 (22.0)	109 489 (10.9)	
2	308 987 (11.1)	259 901 (14.6)	49 086 (4.9)	
3	196 471 (7.1)	169 838 (9.6)	26 633 (2.7)	
4	128 925 (4.6)	112 886 (6.4)	16 039 (1.6)	
≥5	333 788 (12.0)	295 374 (16.6)	38 414 (3.8)	
Differential ED distance, km^f^				
0-8	838 850 (30.2)	767 234 (43.2)	71 616 (7.2)	1.481
9.6-16.0	358 814 (12.9)	306 881 (17.3)	51 933 (5.2)	
17.6-32.0	402 248 (14.5)	304 508 (17.1)	97 740 (9.8)	
33.6-64.0	395 302 (14.2)	217 815 (12.3)	177 487 (17.7)	
>64.0	782 350 (28.2)	180 689 (10.2)	601 661 (60.1)	
Complexity level of patient’s closest VA ED				
1a (most complex)	1 448 177 (52.1)	883 815 (49.7)	564 362 (56.4)	0.188
1b	562 097 (20.2)	393 666 (22.2)	168 431 (16.8)	
1c	431 886 (15.5)	298 825 (16.8)	133 061 (13.3)	
2	285 369 (10.3)	170 616 (9.6)	114 753 (11.5)	
3 (least complex)	50 035 (1.8)	30 205 (1.7)	19 830 (2.0)	

Abbreviations: CDW, Corporate Dara Warehouse; ED, emergency department; EHR, electronic health record; OMOP, Observational Medical Outcomes Partnership; SMD, standardized mean difference; VA, US Department of Veterans Affairs.

^a^ Derived from EHR data from the VA CDW transformed into the OMOP common data model.

^b^ The determination logic prioritizes EHR-reviewed sex and/or gender values across all sources with the most frequent value used. When the method of data entry is unknown or there is no manual review, the OMOP uses the most frequent value from VA Vital Status and Patient data tables.

^c^ Acquired through self-report, proxy, and the enrollment coordinator or clerk, with responses entered into the medical record. OMOP uses a logic and cleans the data, prioritizing self-reported values.

^d^ High disability defined as VA priority groups 1 and 4; low to moderate disability, VA priority groups 2, 3, and 6; low income, VA priority group 5; and no disability, VA priority groups 7 and 8.

^e^ VA risk adjustment measure, where greater than 1.00 reflects greater-than-average health care costs.

^f^ Absolute difference in the linear distance between the patient’s closest VA ED and the patient’s residence and the linear distance between the patient’s closest community ED and the patient’s residence.

Two-thirds of ED visits (1 857 296 [66.9%]) were made by White veterans. Slightly more than half of all visits were made by individuals aged 65 years or older (1 445 666 [52.0%]), more than half were by those with high disability ratings (1 539 062 [55.4%]), and 296 687 (10.7%) were by veterans experiencing housing instability. Most visits (2 643 910 [95.2%]) were by patients who had used VA primary care in the prior year.

Veterans using community EDs were more likely to reside in rural areas (448 712 [44.9%] vs 384 844 [21.7%]), to be White (760 542 [76.0%] vs 1 096 754 [61.7%]), and to be slightly older (mean [SD] age, 63.7 [16.0] vs 62.0 [15.8] years) compared with those using VA EDs. Most veterans sought care at the closest facility. Among ED visits made by veterans who had differential ED distances of 8.0 km or less (n = 838 850), 767 234 visits (91.5%) occurred at a VA ED compared with 71 616 (8.5%) occurring in the community. In contrast, among ED visits made by veterans who had ED differential distances greater than 64.0 km (n = 782 350), 601 661 visits (76.9%) occurred in the community setting, compared with only 180 689 (23.1%) occurring at a VA ED. eTable 1 in Supplement 1 provides characteristics for models 2 to 4.

### Factors Associated With Community ED Use

#### Model 1: All ED Visits

In the full cohort, female (adjusted odds ratio [AOR], 1.10; 95% CI, 1.08-1.11) and unhoused (AOR, 1.13; 95% CI, 1.11-1.15) veterans had higher odds of community ED use. Asian (AOR, 0.64; 95% CI, 0.61-0.67), Black (AOR, 0.76; 95% CI, 0.76-0.77), and Hispanic (AOR, 0.73; 95% CI, 0.72-0.74) veterans had lower odds compared with their non-Hispanic White counterparts. Veterans in rural areas were also less likely to use community EDs than urban veterans (AOR, 0.88; 95% CI, 0.88-0.89).

Geographic proximity was associated with ED location. Veterans whose differential ED distance was greater than 64.0 km had markedly higher odds of community ED use (AOR, 16.20; 95% CI, 15.96-16.44). Even a 9.6- to 16.0-km difference was associated with increased odds of community ED use (AOR, 1.58; 95% CI, 1.56-1.61).

Prior community ED use was among the factors associated with greater odds of future use. Compared with no prior community ED visits, 1 prior visit was associated with greater odds of subsequent ED use (AOR, 3.47; 95% CI, 3.43-3.51), with progressively higher odds for 2 and greater numbers of visits (≥5 prior visits: AOR, 17.87; 95% CI, 17.14-18.63) (Figure and eTable 2 in Supplement 1). AME analysis showed that 1 prior community ED visit was associated with increased probability of future community ED use of 15.73 (95% CI, 15.59-15.88) percentage points. A differential ED distance greater than 64.0 km was associated with increased probability of 39.94 (95% 39.74-40.14) percentage points compared with 1.01 (95% CI, 0.86-1.16) and 1.37 (95% CI 1.16-1.59) percentage points for female sex and housing instability, respectively.

**Figure.**
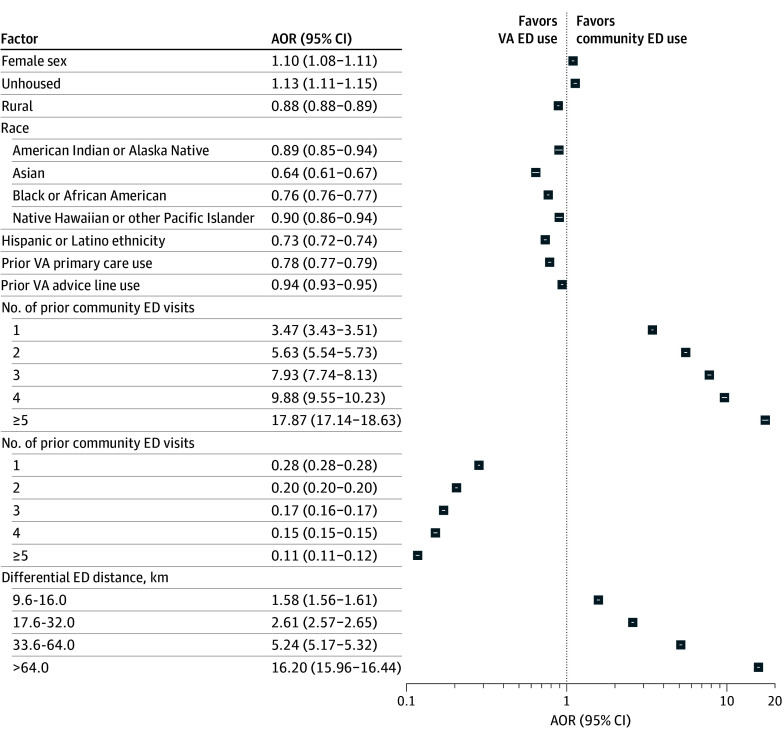
Forest Plot of Selected Adjusted Odds Ratios (AORs) of Patient Factors Associated With Community Emergency Department (ED) Use Odds ratios were adjusted for patient, encounter, and facility characteristics and temporal variables (month and day of week). *P* < .001 for all AORs. VA indicates US Department of Veterans Affairs.

In contrast, prior VA ED use was associated with lower odds of community ED visits (1 prior ED visit: AOR, 0.28 [95% CI, 0.28-0.28]; ≥5 visits: AOR, 0.11 [95% CI, 0.11-0.12]). Prior VA primary care (AOR, 0.78; 95% CI, 0.77-0.79) and VA nurse advice line use (AOR, 0.94; 95% CI, 0.93-0.95) were also associated with lower community ED use.

ED diagnoses were associated with setting. Life-threatening conditions, including cardiac arrest and ventricular fibrillation (AOR, 84.57; 95% CI, 65.47-109.25), poisoning by drugs (AOR, 43.84; 95% CI, 39.01-49.26), septicemia (AOR, 27.99; 95% CI, 26.80-29.24), and hemorrhagic stroke (AOR, 17.94; 95% CI, 15.54-20.71), were associated with greater odds of community ED use. Traumatic conditions, such as internal organ injury (AOR, 48.87; 95% CI, 40.62-58.79), hip fracture (OR, 29.20; 95% CI, 26.43-32.27), and traumatic brain injury or concussion (AOR, 14.82; 95% CI, 13.77-15.95), were also associated with higher odds of community ED use. Conversely, visits for lower-acuity diagnoses, such as uncomplicated diabetes (AOR, 0.52; 95% CI, 0.46-0.60), sinusitis (AOR, 0.83; 95% CI, 0.78-0.87), and low back pain (AOR, 0.78; 95% CI, 0.76-0.81), were more likely to occur at VA EDs (Table 2). Veterans whose nearest VA ED was a low-complexity (level 3) facility had higher odds of using a community ED compared with those near a level 1a facility (OR, 1.26; 95% CI, 1.22-1.29).

**Table 2.** AORs and AMEs for Diagnoses Associated With the Highest and Lowest Odds of Community ED Use

Clinical classification category^a^	AOR (95% CI)^b^	AME (95% CI)^c^
Other specified complications in pregnancy	103.40 (83.96 to 127.34)	58.69 (56.63 to 60.76)
Cardiac arrest and ventricular fibrillation	84.57 (65.47 to 109.25)	56.62 (53.89 to 59.35)
Aspiration pneumonitis	53.06 (43.48 to 64.76)	51.29 (48.87 to 53.71)
Internal organ injury	48.87 (40.62 to 58.79)	50.28 (48.00 to 52.56)
Poisoning by drugs	43.84 (39.01 to 49.26)	48.92 (47.45 to 50.39)
Early, first, or unspecified trimester hemorrhage	40.66 (32.53 to 50.83)	47.96 (45.10 to 50.82)
Fracture of the neck of the femur (hip)	29.20 (26.43 to 32.27)	43.61 (42.28 to 44.94)
Septicemia	27.99 (26.80 to 29.24)	43.04 (42.47 to 43.61)
Complication of internal orthopedic device or implant	22.87 (18.37 to 28.48)	40.30 (37.29 to 43.30)
Acute hemorrhagic cerebrovascular disease	17.94 (15.54 to 20.71)	36.94 (34.95 to 38.93)
Encounter for observation and examination for conditions ruled out^d^	0.65 (0.60 to 0.70)	−4.42 (−5.13 to −3.71)
Fungal infections	0.64 (0.57 to 0.71)	−4.53 (−5.58 to −3.48)
Exposure, encounters, screening or contact with infectious disease	0.60 (0.57 to 0.62)	−5.22 (−5.63 to −4.81)
Medical examination and/or evaluation	0.53 (0.48 to 0.58)	−6.31 (−7.17 to −5.45)
Diabetes without complication	0.52 (0.46 to 0.60)	−6.47 (−7.72 to −5.23)
Sexually transmitted infections (excluding HIV and hepatitis)	0.46 (0.40 to 0.53)	−7.54 (−8.77 to −6.31)
Other specified status^e^	0.28 (0.26 to 0.30)	−11.77 (−12.25 to −11.29)
Personal and/or family history of disease	0.25 (0.21 to 0.30)	−12.61 (−13.89 to −11.33)
Injury, sequela	0.11 (0.09 to 0.13)	−18.07 (−19.11 to −17.02)
Other specified encounters and counseling^f^	0.08 (0.08 to 0.09)	−19.37 (−19.76 to −18.98)

Abbreviations: AME, average marginal effect; AOR, adjusted odds ratio; ED, emergency department.

^a^ *International Statistical Classification of Diseases, Tenth Revision, Clinical Modification* primary diagnosis from the ED visit reported using the Agency for Healthcare Research and Quality Clinical Classification Software Refined categories (version 2022.1).

^b^ Adjusted for patient, encounter, and facility characteristics and temporal variables (month and day of week). *P* < .001 for all AORs.

^c^ Calculated as the average of all the observation-specific marginal effects. *P* < .001 for all AMEs.

^d^ Observation and examination for conditions excluding infectious diseases, neoplasm, and mental disorders. Category typically includes encounters where conditions are ultimately ruled out.

^e^ Z diagnosis codes that describe factors influencing health status and contact with health services outside acute illness or injury.

^f^ Z diagnosis codes for encounters related to counseling, supervision, or other specified factors influencing health status.

#### Model 2: ED Visits Among Veterans With Similar Geographic Access

Among veterans whose differential ED distances were 8.0 km or less, associations generally mirrored those in model 1, with some differences in magnitude of the odds (eTable 2 in Supplement 1). Female (AOR, 1.12; 95% CI, 1.08-1.15), unhoused (AOR, 1.17; 95% CI, 1.13-1.21), and younger (18-44 years: AOR, 1.18; 95% CI, 1.14-1.22) veterans had higher odds of community ED use. While race and ethnicity continued to be associated with lower odds of community ED use among racial and ethnic minority veterans, the magnitude of differences was slightly attenuated in this geographically restricted sample. Prior VA primary care use (AOR, 0.65; 95% CI, 0.62-0.68) and nurse advice line use (AOR, 0.73; 95% CI, 0.71-0.76) were again associated with lower likelihood of community ED use. AMEs showed that these factors were associated with fewer community ED visits of −2.97 (95% CI, −3.29 to −2.64) and −1.76 (95% CI, −1.93 to −1.59) percentage points, respectively. The primary diagnosis for the ED visit and prior community ED use remained the factors associated with the greatest odds of community ED use.

#### Model 3: ED Visits Among Veterans With Chest Pain

Model 3 focused on ED visits for chest pain among veterans whose differential ED distances were 8.0 km or less. Prior health care use remained associated with ED setting. Veterans with prior VA primary care use had significantly lower odds of community ED use (AOR, 0.60; 95% CI, 0.51-0.72), as did those who used the VA nurse advice line (AOR, 0.85; 95% CI, 0.74-0.96), with lower odds than those observed in the full cohort (eTable 2 in Supplement 1).

Black veterans had higher odds of VA ED use compared with White veterans (AOR, 0.77; 95% CI, 0.71-0.85). Notably, a new association emerged in this subgroup: veterans who had a VA mental health care visit in the prior year had significantly higher odds of using a community ED (AOR, 1.14; 95% CI, 1.04-1.25).

#### Model 4: ED Visits Among Veterans With No Prior ED Use

Model 4 focused on ED visits by veterans with no ED use in the prior 12 months. Geographic proximity remained associated with increased odds of community ED use (eTable 2 in Supplement 1). Veterans with greater differential ED distances had significantly higher odds of using community care (>64.0 km: AOR, 49.67; 95% CI, 48.64-50.72). Primary diagnosis was associated with ED site, with acute and injury-related conditions associated with greater likelihood of community ED use. Race and ethnicity were also associated with ED setting, with American Indian or Alaska Native (AOR, 0.87; 95% CI, 0.82-0.93), Asian (AOR, 0.64; 95% CI, 0.60-0.68), Black (AOR, 0.66; 95% CI, 0.65-0.67), and Native Hawaiian or Other Pacific Islander (AOR, 0.86; 95% CI, 0.81-0.92) veterans more likely to use VA EDs than community EDs after adjustment for other factors.

## Discussion

In this national cross-sectional study of 2 777 564 million ED visits, we identified factors associated with veterans’ use of community vs VA EDs. Community ED use was associated with prior patterns of care, clinical presentation, and demographic characteristics. Veterans with recent VA ED or primary care use were significantly less likely to use community EDs, suggesting that ongoing VA engagement may be associated with site of care decisions. These patterns highlight differences in which veterans are more likely to use community vs VA EDs, raising questions about how reliance on each setting may vary across populations. As the VA continues to rely on both VA and community options to meet emergency care needs, characterizing these patterns is important for informing delivery models and supporting equitable access. Geographic proximity was, as expected, associated with some of the highest odds of community ED use. A total of 28.2% of visits involved a differential ED distance greater than 64.0 km, and this group had more than 16 times the odds of using community care. These findings suggest that community care is functioning as intended by extending access where VA care is geographically limited. However, community ED use may be associated with increased risk of fragmented care.^11^ Maintaining access to VA EDs for veterans is critical to ensure continuity and avoid disruptions in follow-up and specialty services. Strategies to balance access and integration could include expanding the VA’s tele-emergency care program,^12^ improving coordination between VA and community clinicians,^13^ and enhancing transportation support.^14^ Increasing the availability of urgent or same-day care at VA clinics may also help meet emergency needs in areas without nearby VA EDs.^15^

Veterans with life-threatening or trauma-related conditions, including cardiac arrest, drug poisoning, and internal injuries, were substantially more likely to seek care at community EDs. These patterns likely reflect the urgency of symptoms and the need to access the closest available facility. Conversely, lower-acuity conditions, such as uncomplicated diabetes or low back pain, were more commonly treated in VA EDs. These patterns underscore the importance of proximity in emergencies and reinforce prior qualitative findings suggesting that veterans may prefer VA care when time and safety allow but prioritize proximity in high-acuity scenarios.^6^ Rapid follow-up and care coordination after community ED visits could help minimize disruptions in care and reconnect veterans to VA-based services.

Prior community ED use was associated with some of the greatest odds of subsequent use. Veterans with even a single community ED visit in the prior year were more likely to return, with the odds increasing with each additional visit. These patterns may reflect familiarity, perceived convenience, or perceptions of quality and suggest a reinforcing cycle of community reliance. Conversely, frequent users of VA EDs were markedly less likely to seek care at community EDs, highlighting the critical role of continuity of care within the VA system. Veterans who engage early and regularly with VA services are likely more familiar with VA resources and may develop stronger relationships with VA health care clinicians, fostering trust and increasing their likelihood of seeking future care within the VA system.^6,16^

We observed consistent racial and ethnic differences in ED site of care. After adjusting for geography, clinical need, and utilization history, Asian, Black, and Hispanic veterans had lower odds of community ED use compared with White veterans. These differences persisted across subgroups, including veterans with similar geographic access or similar diagnoses. While this finding may reflect greater trust in VA care among racial and ethnic minoritized veterans, it may also signal barriers to accessing community services, including administrative hurdles, lower awareness of eligibility, or prior negative experiences.^5,17,18,19^ Further research is needed to determine whether these patterns reflect true preferences, differential access, or systemic inequities. As community care continues to expand, ensuring equitable access for racial and ethnic minority veterans will be critical.

Although rural veterans accounted for a large share of community ED visits overall, their adjusted odds of community ED use were lower than those of urban veterans. This may reflect greater willingness to travel for VA care or stronger familiarity with the VA system. It is also possible that the association with rural residence is correlated with geographic distance, which was explicitly controlled for in our models. Nonetheless, tailored services, such as tele-emergency care, transportation support, expanded outreach about local VA options, and investments in rural infrastructure (eg, co-located urgent care services within clinics), may still be particularly beneficial in rural areas, where both VA and community resources can be limited. Veterans living near low-complexity VA EDs had higher odds of seeking emergency care in the community even after accounting for distance. This suggests that perceptions or realities of limited capability at local VA EDs are associated with care-seeking behavior. Strengthening services at lower-complexity VA sites, clarifying available services, and expanding access to specialty expertise through telemedicine partnerships^20^ may increase confidence in VA EDs and reduce community reliance.^21^

### Limitations

This study has limitations. First, administrative data may be subject to miscoding or variation in how diagnoses and services are recorded. Second, although models adjusted for multiple patient and encounter factors, findings should be interpreted as associations and not causal effects or predictors. Third, unmeasured factors, such as veteran preferences, emergency medical services routing decisions, or satisfaction with care, were not captured in this analysis. In particular, arrival method (eg, ambulance, walk-in) was not available in national VA or community ED data, although it likely plays a role in influencing ED setting and may be related to other factors in the models.

## Conclusions

In this cross-sectional study, community ED use among veterans was associated with geographic access, prior care patterns, and diagnosis acuity, highlighting the central role of these factors in community ED use among veterans. Partnerships with the VA’s Office of Integrated Veteran Care and national emergency medicine leadership could enhance veteran education, outreach after community ED visits, and broader implementation of tele-emergency care. Supporting lower-complexity VA EDs and expanding virtual care services may increase veterans’ access to VA acute care, particularly for veterans who reside far from a VA ED.

## Notes

Supplement 1. **eTable 1.** Characteristics of VA ED Visits and Community ED Visits Purchased by VA, Fiscal Year 2022 (Models 2-4) **eTable 2.** Adjusted Odds Ratios and 95% CIs for Patient and Facility Factors Associated With Community ED Use (Models 1-4)

Supplement 2.
Data Sharing Statement
