# Supplementary material for: Factors Associated With Veterans Use of Community vs VA Emergency Departments
Source: JAMA Netw Open. 2025 Dec 8;8(12):e2543062. doi: 10.1001/jamanetworkopen.2025.43062 (PMC12687090; doi:10.1001/jamanetworkopen.2025.43062)
Supplement: Supplement 1. — eTable 1. Characteristics of VA ED Visits and Community ED Visits Purchased by VA, Fiscal Year 2022 (Models 2-4) eTable 2. Adjusted Odds Ratios and 95% CIs for Patient and Facility Factors Associated With Community ED Use (Models 1-4) [file jamanetwopen-e2543062-s001.pdf]

Supplementary Online Content

Vashi AA, Asch SM, Urech T, We S, Tran LD. Factors associated with veterans use of community vs VA emergency departments. *JAMA Netw Open.* 2025;8(12):e2543062. doi:10.1001/jamanetworkopen.2025.43062

eTable 1. Characteristics of VA ED Visits and Community ED Visits Purchased by VA, Fiscal Year 2022 (Models 2-4)

eTable 2. Adjusted Odds Ratios and 95% CIs for Patient and Facility Factors Associated With Community ED Use (Models 1-4)

This supplementary material has been provided by the authors to give readers additional information about their work.

© 2025 Vashi AA et al. *JAMA Network Open*.

eTable 1. Characteristics of VA ED Visits and Community ED Visits Purchased by VA, Fiscal Year 2022 (Models 2-4)

	Distance to Nearest VA and Community ED Differed by ≤ 5 miles (Model 2)				Distance to Nearest VA and Community ED Differed by ≤ 5 miles + Chest Pain Dx (Model 3)				No Prior ED Use in the 12 Months Preceding the ED Visit (Model 4)			
**No. of ED visits**	**Overall**	**VA**	**Comm**	**SMD**	**Overall**	**VA**	**Comm**	**SMD**	**Overall**	**VA**	**Comm**	**SMD**
**Characteristic, %**	838,850	767,234	71,616		32,279	28,641	3,638		849,477	483,587	365,890	
Age group				0.042				0.165				0.153
18-44	16.1	16.0	17.3		15.0	14.5	18.6		18.6	19.8	17.0	
45-64	32.0	32.1	30.6		34.8	34.3	38.1		30.2	32.4	27.4	
>/=65	52.0	52.0	52.0		50.3	51.2	43.3		51.2	47.9	55.5	
Female sex	11.3	11.3	11.9	0.018	11.5	11.3	12.8	0.047	11.2	11.8	10.4	0.043
Race				0.167				0.186				0.341
AIAN	0.8	0.8	1.0		0.8	0.8	1.1		0.8	0.8	0.9	
Asian	0.7	0.7	0.6		0.7	0.7	0.5		0.9	1.2	0.5	
Black or AA	34.9	35.5	28.2		38.0	38.9	30.8		21.2	26.7	13.9	
NHOPI	0.8	0.8	0.7		0.9	0.9	0.8		0.8	0.9	0.7	
White	57.0	56.4	64.0		54.1	53.1	61.8		69.6	63.7	77.4	
Unknown	5.8	5.8	5.6		5.6	5.7	5.0		6.6	6.7	6.4	
Ethnicity				0.101				0.076				0.135
Hispanic/ Latino	8.7	8.9	6.3		9.6	9.8	7.7		7.7	9.2	5.7	
Not Hispanic/ Latino	89.2	89.0	91.6		88.5	88.3	90.5		89.6	88.2	91.5	
Unknown	2.1	2.1	2.1		1.9	2.0	1.7		2.7	2.6	2.8	
Unhoused	15.5	14.9	21.6	0.172	15.5	14.7	21.7	0.182	5.1	5.9	4.1	0.084
Rural location	9.6	9.6	10.1	0.019	9.2	9.1	10.1	0.034	33.8	23.7	47.1	0.506
VA priority group[a]				0.116				0.127				0.040
Highly disabled	51.9	51.5	56.1		52.7	52.3	56.5		52.1	52.0	52.3	
Low/mod disability	18.1	18.3	16.1		17.8	18.0	16.3		19.4	19.6	19.1	
Low income	19.8	19.8	19.8		19.7	19.7	20.2		16.2	15.8	16.9	
Nondisabled	10.3	10.5	8.0		9.7	10.1	7.0		12.2	12.6	11.7	
Nosos risk score				0.125				0.072				0.066
< 1.00	31.5	31.6	30.4		29.6	29.4	30.6		54.7	53.6	56.3	
1.00-1.99	34.3	34.7	30.1		34.1	34.5	31.1		33.3	33.7	32.8	
>/= 2.00	34.2	33.8	39.5		36.3	36.1	38.3		11.9	12.7	10.9	

© 2025 Vashi AA et al. *JAMA Network Open*.

eTable 1 continued. Characteristics of VA ED Visits and Community ED Visits Purchased by VA, Fiscal Year 2022 (Models 2-4)

	Distance to Nearest VA and Community ED Differed by ≤ 5 miles (Model 2)				Distance to Nearest VA and Community ED Differed by ≤ 5 miles + Chest Pain Dx (Model 3)				No Prior ED Use in the 12 Months Preceding the ED Visit (Model 4)			
	**Overall**	**VA**	**Comm**	**SMD**	**Overall**	**VA**	**Comm**	**SMD**	**Overall**	**VA**	**Comm**	**SMD**
**Characteristic, %**	838,850	767,234	71,616		32,279	28,641	3,638		849,477	483,587	365,890	
Prior VA primary care use[b]	95.7	95.9	93.1	0.124	96.7	97.0	94.5	0.124	92.8	93.2	92.3	0.035
Prior VA mental health use[b]	52.4	52.0	56.6	0.092	54.8	53.9	62.2	0.169	37.9	39.7	35.6	0.083
Prior VA advice line use[c]	10.9	11.2	8.0	0.111	10.6	10.7	9.3	0.048	14.4	14.9	13.6	0.037
No. of prior community ED visits[b]				0.869				0.867				n/a
0	85.3	88.3	52.7		82.7	86.7	51.0		n/a	n/a	n/a	
1	8.4	7.3	19.7		9.1	8.0	18.0		n/a	n/a	n/a	
2	2.6	2.0	9.3		3.0	2.2	9.0		n/a	n/a	n/a	
3	1.2	0.9	5.2		1.4	0.9	5.4		n/a	n/a	n/a	
4	0.7	0.4	3.1		0.8	0.6	2.8		n/a	n/a	n/a	
≥5	1.8	1.0	9.9		3.0	1.6	13.9		n/a	n/a	n/a	
No. of prior VA ED visits[b]				0.286				0.249				n/a
0	26.1	25.0	38.0		26.4	25.3	35.4		n/a	n/a	n/a	
1	20.8	21.0	18.4		19.4	19.7	17.6		n/a	n/a	n/a	
2	14.9	15.2	12.0		14.5	14.8	11.7		n/a	n/a	n/a	
3	10.3	10.5	7.9		9.8	10.1	7.6		n/a	n/a	n/a	
4	7.1	7.3	5.5		7.0	7.3	4.8		n/a	n/a	n/a	
≥5	20.8	21.1	18.2		22.9	22.9	22.9		n/a	n/a	n/a	
Differential ED distance, km[d]				n/a				n/a				1.530
0-8	n/a	n/a	n/a		n/a	n/a	n/a		23.4	37.6	4.6	
9.6-16.0	n/a	n/a	n/a		n/a	n/a	n/a		11.8	17.4	4.3	
17.6-32.0	n/a	n/a	n/a		n/a	n/a	n/a		14.7	19.0	9.1	
33.6-64.0	n/a	n/a	n/a		n/a	n/a	n/a		15.7	14.1	17.7	
>64.0	n/a	n/a	n/a		n/a	n/a	n/a		34.4	11.8	64.3	
Facility complexity level of patient's closest VA ED				0.133				0.187				0.186
1a (most complex)	41.7	41.9	39.9		43.9	44.5	39.7		53.7	50.7	57.7	
1b	24.9	25.2	21.6		25.3	25.7	22.6		19.4	21.5	16.8	
1c	18.3	18.2	19.7		17.1	16.9	18.6		14.9	16.6	12.6	

© 2025 Vashi AA et al. *JAMA Network Open*.

2	12.3	12.0	15.0	11.4	10.9	15.5	10.2	9.6	11.0
3 (least complex)	2.8	2.7	3.8	2.3	2.1	3.6	1.7	1.6	1.9

Abbreviations: AA, African American; AIAN, American Indian or Alaska Native; Comm, community; NHOPI, Native Hawaiian or Other Pacific Islander.

[a] Highly disabled defined as VA Priority Groups 1 and 4. Low/moderate disability defined as VA Priority Groups 2, 3, and 6. Low income defined as VA Priority Group 5. Nondisabled defined as VA Priority Groups 7 and 8.

[b] Prior use defined as use in the 12 months preceding the ED visit

[c] Prior use defined as use in the 2 days before the ED visit

[d] Differential ED distance defined as the absolute difference in the linear distance between the patient's closest VA ED and the patient's residence and the linear distance between the patient's closest community ED and the patient's residence. The categories (e.g., 0-5) indicate the range in the mileage difference between the patient's closest VA ED and closest community ED.

© 2025 Vashi AA et al. *JAMA Network Open*.

eTable 2. Adjusted Odds Ratios and 95% CIs for Patient and Facility Factors Associated With Community ED Use (Models 1-4)[a]

	**All ED Visits (Model 1)**				**Distance to Nearest VA and Community ED Differed by ≤ 5 miles (Model 2)**				**Distance to Nearest VA and Community ED Differed by ≤ 5 miles + Chest Pain Dx (Model 3)**				**No Prior ED Use in the 12 Months Preceding the ED Visit (Model 4)**			
**Number of ED Visits**	2,777,564				838,850				32,279				849,477			
**Predictor**	**OR**	**95% CI**		**P**	**OR**	**95% CI**		**P**	**OR**	**95% CI**		**P**	**OR**	**95% CI**		**P**
Age group (Ref: 65+)																
18-44	1.01	0.99	1.02	0.264	1.18	1.14	1.22	<0.001	1.32	1.17	1.50	<0.001	0.96	0.95	0.98	<0.001
45-64	0.95	0.94	0.96	<0.001	1.03	1.01	1.06	0.011	1.19	1.09	1.31	<0.001	0.90	0.88	0.91	<0.001
Female sex (Ref: Male sex)	1.10	1.08	1.11	<0.001	1.12	1.08	1.15	<0.001	1.09	0.97	1.22	0.159	1.13	1.11	1.15	<0.001
Unhoused (Ref: Housed)	1.13	1.11	1.15	<0.001	1.17	1.13	1.21	<0.001	1.06	0.94	1.21	0.329	1.15	1.12	1.19	<0.001
Rural (Ref: Urban)	0.88	0.88	0.89	<0.001	0.97	0.93	1.01	0.146	1.00	0.88	1.15	0.968	0.87	0.86	0.88	<0.001
VA Priority Group (Ref: Highly disabled)																
Low/moderate disability	0.92	0.91	0.93	<0.001	0.84	0.82	0.87	<0.001	0.89	0.79	1.00	0.042	0.89	0.88	0.90	<0.001
Low income	1.01	0.99	1.02	0.403	0.89	0.86	0.91	<0.001	0.98	0.87	1.10	0.704	0.99	0.97	1.01	0.284
Nondisabled	0.90	0.89	0.92	<0.001	0.79	0.77	0.82	<0.001	0.77	0.66	0.89	0.001	0.89	0.87	0.90	<0.001
Race (Ref: White)																
AIAN	0.89	0.85	0.94	<0.001	0.98	0.85	1.13	0.816	1.21	0.78	1.87	0.388	0.87	0.82	0.93	<0.001
Asian	0.64	0.61	0.67	<0.001	0.77	0.69	0.87	<0.001	0.84	0.51	1.40	0.506	0.64	0.60	0.68	<0.001
Black or AA	0.76	0.76	0.77	<0.001	0.83	0.81	0.85	<0.001	0.77	0.71	0.85	<0.001	0.66	0.65	0.67	<0.001
NHOPI	0.90	0.86	0.94	<0.001	0.91	0.81	1.01	0.083	0.76	0.51	1.14	0.187	0.86	0.81	0.92	<0.001
Unknown	0.98	0.96	1.00	0.022	0.97	0.93	1.01	0.155	0.92	0.76	1.11	0.378	0.96	0.93	0.98	<0.001
Ethnicity (Ref: Not Hispanic or Latino)																
Hispanic or Latino	0.73	0.72	0.74	<0.001	0.76	0.73	0.79	<0.001	0.87	0.75	1.01	0.063	0.67	0.65	0.68	<0.001
Unknown	1.04	1.01	1.06	0.007	1.00	0.94	1.07	0.918	0.96	0.71	1.28	0.776	1.07	1.03	1.12	<0.001
Nosos risk score (Ref: < 1.00)																
1.00-1.99	0.89	0.88	0.90	<0.001	0.96	0.93	0.98	0.002	0.98	0.88	1.09	0.705	0.84	0.83	0.85	<0.001
≥ 2.00	0.87	0.86	0.88	<0.001	0.99	0.96	1.02	0.383	0.92	0.82	1.05	0.211	0.73	0.72	0.75	<0.001
Prior VA primary care use	0.78	0.77	0.79	<0.001	0.65	0.62	0.68	<0.001	0.60	0.51	0.72	<0.001	0.78	0.76	0.80	<0.001
Prior VA mental health use	1.04	1.03	1.05	<0.001	1.03	1.00	1.05	0.025	1.14	1.04	1.25	0.005	1.01	1.00	1.03	0.089
Prior VA advice line use	0.94	0.93	0.95	<0.001	0.73	0.71	0.76	<0.001	0.85	0.74	0.96	0.011	0.87	0.86	0.88	<0.001

© 2025 Vashi AA et al. *JAMA Network Open*.

eTable 2 continued. Adjusted Odds Ratios and 95% CIs for Patient and Facility Factors Associated with Community ED Use, Models 1-4[a]

	All ED Visits (Model 1)				Distance to Nearest VA and Community ED Differed by ≤ 5 miles (Model 2)				Distance to Nearest VA and Community ED Differed by ≤ 5 miles + Chest Pain Dx (Model 3)				No Prior ED Use in the 12 Months Preceding the ED Visit (Model 4)			
**Number of ED visits**	2,777,564				838,850				32,279				849,477			
**Predictor**	**OR**	**95% CI**		**P**	**OR**	**95% CI**		**P**	**OR**	**95% CI**		**P**	**OR**	**95% CI**		**P**
Number of prior community ED visits (Ref: none)																
1	3.47	3.43	3.51	<0.001	5.02	4.89	5.15	<0.001	4.69	4.21	5.22	<0.001	n/a	n/a		n/a
2	5.63	5.54	5.73	<0.001	9.22	8.87	9.59	<0.001	9.33	7.98	10.91	<0.001	n/a	n/a		n/a
3	7.93	7.74	8.13	<0.001	12.65	11.97	13.38	<0.001	12.83	10.31	15.96	<0.001	n/a	n/a		n/a
4	9.88	9.55	10.23	<0.001	16.41	15.23	17.68	<0.001	11.89	9.06	15.62	<0.001	n/a	n/a		n/a
≥5	17.87	17.14	18.63	<0.001	26.76	24.54	29.18	<0.001	26.13	21.12	32.32	<0.001	n/a	n/a		n/a
Number of prior VA ED visits (Ref: none)																
1	0.28	0.28	0.28	<0.001	0.54	0.52	0.55	<0.001	0.58	0.52	0.64	<0.001	n/a	n/a		n/a
2	0.20	0.20	0.20	<0.001	0.43	0.42	0.45	<0.001	0.47	0.41	0.53	<0.001	n/a	n/a		n/a
3	0.17	0.16	0.17	<0.001	0.37	0.35	0.38	<0.001	0.40	0.34	0.46	<0.001	n/a	n/a		n/a
4	0.15	0.15	0.15	<0.001	0.34	0.33	0.36	<0.001	0.31	0.26	0.37	<0.001	n/a	n/a		n/a
≥5	0.11	0.11	0.12	<0.001	0.23	0.22	0.23	<0.001	0.27	0.23	0.31	<0.001	n/a	n/a		n/a
Differential ED distance, km (Ref: 0-8)																
9.6-16.0	1.58	1.56	1.61	<0.001	n/a	n/a		n/a	n/a	n/a		n/a	2.08	2.03	2.13	<0.001
17.6-32.0	2.61	2.57	2.65	<0.001	n/a	n/a		n/a	n/a	n/a		n/a	4.08	3.99	4.17	<0.001
33.6-64.0	5.24	5.17	5.32	<0.001	n/a	n/a		n/a	n/a	n/a		n/a	10.88	10.65	11.11	<0.001
>64.0	16.20	15.96	16.44	<0.001	n/a	n/a		n/a	n/a	n/a		n/a	49.67	48.64	50.72	<0.001
Facility complexity level of patient's closest VA ED (ref: 1a - most complex)																
1b	0.91	0.90	0.92	<0.001	0.97	0.94	0.99	0.011	1.15	1.03	1.27	0.01	0.84	0.83	0.86	<0.001
1c	0.89	0.87	0.90	<0.001	1.14	1.10	1.17	<0.001	1.31	1.17	1.47	<0.001	0.76	0.75	0.78	<0.001
2	1.05	1.03	1.06	<0.001	1.14	1.11	1.18	<0.001	1.48	1.30	1.68	<0.001	0.96	0.94	0.98	<0.001
3 (least complex)	1.26	1.22	1.29	<0.001	1.32	1.25	1.40	<0.001	1.76	1.41	2.20	<0.001	1.17	1.12	1.23	<0.001

Abbreviations: AA, African American; AIAN, American Indian or Alaska Native; NHOPI, Native Hawaiian or Other Pacific Islander.

© 2025 Vashi AA et al. *JAMA Network Open*.

[a] Odds ratios were adjusted for patient, encounter and facility characteristics and temporal variables (month and day of week).

© 2025 Vashi AA et al. *JAMA Network Open*.
