# Supplementary material for: Factors Associated With Veterans Use of Community vs VA Emergency Departments
Source: JAMA Netw Open. 2025 Dec 8;8(12):e2543062. doi: 10.1001/jamanetworkopen.2025.43062 (PMC12687090; doi:10.1001/jamanetworkopen.2025.43062)
Supplement: Supplement 2. — Data Sharing Statement [file jamanetwopen-e2543062-s002.pdf]

Data Sharing Statement

Vashi. Factors Associated With Veterans Use of Community vs VA Emergency Departments. *JAMA Netw Open*. Published December 08, 2025. doi:10.1001/jamanetworkopen.2025.43062

Data
Data available: No
